# Neonatal Septic Arthritis of the Hip: A Case Report

**DOI:** 10.7759/cureus.42738

**Published:** 2023-07-31

**Authors:** Mohammad N Almatrafi, Mohammed A Almatrafi, Huda M Qronfla, Majed T Osaylan, Salem A Bajuifer

**Affiliations:** 1 Department of Orthopaedics, Alnoor Specialist Hospital, Mecca, SAU; 2 Department of Orthopaedic Surgery, Alnoor Specialist Hospital, Mecca, SAU; 3 Department of Medicine, King Abdulaziz University Faculty of Medicine, Jeddah, SAU

**Keywords:** surgery, pediatric orthopedic surgery, neonate, hip joint, septic arthritis

## Abstract

Neonatal septic arthritis is a bacterial infection that, if not identified and treated right away, can affect human joints and result in osteonecrosis, joint destruction, and permanent abnormalities. Around 0.3 out of every 1,000 live births are impacted by septic arthritis worldwide. Although there is very little available data on the newborn population, we present a novel case of neonatal hip septic arthritis that was presented to our hospital and was successfully treated with surgical irrigation and debridement through arthrotomy with the Smith-Petersen approach in addition to antibiotics, despite its early vague presentation and lack of complications. Thus, it is crucial to clinically and radiologically evaluate neonates presenting with obscure symptoms and signs to prevent future disabilities and complications resulting from septic arthritis.

## Introduction

Neonatal septic arthritis (SA) is a bacterial infection that causes the destruction of a joint and reaches human joints through hematogenous spread or directly through trauma that emergently requires an orthopedic consultation and intervention [1,2]. In particular, it is commonly spread by a hematogenous spread in newborns due to the abundant vascular supply and absence of synovial basement membrane in their joints [3]. This infection tends to affect mainly lower limb joints, including the hip, knee, or ankle, particularly in the pediatric age group, in which it commonly affects the hip and knee by 32%-39% and 26%-47%, respectively, by *Staphylococcus aureus* pathogen, while additional bacteria that have been found in culture include *Klebsiella pneumoniae*, Group B *Streptococci*, *Escherichia coli*, *Enterobacter sp.*, *Kingella kingae*, and *Candida spp.* [1,2,4,5]. Moreover, SA globally affects around 0.3 out of every 1,000 live births [6]. The United States of America (USA) carries the highest incidence among pediatrics in those from 0 to four years old [7]; however, it is rarely reported to affect neonates or newborns less than 28 days old [4]. Also, it affects 0.6 out of every 1,000 live births in India [6]. Because it remains undiagnosed in the early stages due to the lack of symptoms, it can lead to grave consequences, including the newborn's death [3]. Also, because newborns have an immature immune system, the disease has a tendency to spread quickly and cause a number of dreadful outcomes, including the destruction of articular cartilage and ossification centers, sepsis, osteomyelitis, meningitis, the formation of abscesses in tissue spaces, urinary tract infections, and others, according to the previously published case reports and series that were limited to neonatal age [8].

Therefore, we present a novel case of septic arthritis of the hip joint in a neonate who was investigated and managed surgically with a good prognosis and effective treatment at the Orthopedic Department in Alnoor Specialist Hospital, Makkah, Saudi Arabia, in collaboration with Maternity and Children Hospital, Makkah, Saudi Arabia.

## Case presentation

The patient had a thorough clinical history and physical assessment, and the patient's father provided written informed consent for the use of radiological imaging and to publish this article as a case report, although the training and education unit does not require ethical approval for reporting case reports.

This is the case of a six-day-old baby boy, medically free, a product of a cesarean section (CS) of 38 weeks of gestation with oligohydramnios, who presented with his parents to the emergency department (ED) of our hospital with a painfully limited range of motion of the right hip.

The patient was in his usual state of health until his parents noticed a limitation of movement in his right hip associated with irritability each time they tried to move his right thigh. However, it was not associated with fever, a change in urine amount, color, or smell. Also, there was negative history for ear ripping, runny nose, drooling, cough (dry or productive), stridor/wheezing, and chest infection; and for shivering, seizures, photophobia (turning away from lights), projectile vomiting, and pin-prick rash. His other review of systems was unremarkable. According to his parents, the patient was admitted for one day in the neonatal intensive care unit (NICU) due to oligohydramnios for observations; otherwise, he was medically and surgically free. Regarding his perinatal history, he was a full-term baby boy at 38 weeks of gestation; during pregnancy, the mother did regular follow-ups. There was no history of maternal fever, rash, gestational diabetes mellitus, preeclampsia or eclampsia, or exposure to radiation, smoking, or drugs. The mother was on folic acid during her pregnancy. The antenatal screening ultrasound did not show any abnormality.

He was delivered through CS, and his birth weight was measured at 3.5 kg. After delivery, he cried immediately and had normal color (no cyanosis or pallor) and good muscle tone. However, postnatally, he was admitted to the neonatal intensive care unit (NICU), according to the parents, for one day due to oligohydramnios for observation. Regarding his nutritional status, he was exclusively breastfed. He only received his first vaccine for hepatitis B before discharging post-delivery, and regarding his development, he was up to his age.

Also, there was no similar condition in the family and no family history of musculoskeletal disorders or inherited diseases, neonatal death, or previous unexplained recurrent miscarriages. In the physical examination, the patient was vitally stable when he was first seen in the ED. He looked conscious and was not in respiratory distress. He was apparently not cyanosed, pale, or jaundiced. He has no dysmorphic features, and he has no signs of dehydration. His nutritional status looked good and was environmentally free; he was only connected to the monitor. His head-to-toe examination was unremarkable, except upon local examination of his right hip, where the right limb rested in a position of slight flexion, abduction, and external rotation. Also, redness was noticed, and heat was felt upon palpation as well as irritability while moving the right hip passively. However, a distal pulse was intact, the contralateral hip was compared, and it was normal.

Regarding investigations, he had been extensively investigated for a complete blood count (CBC), which revealed the following: leucocytes (WBC): 16.23 x 10^9/L; automated neutrophils: 49.7% (reference range: 50%-70%); automated lymphocytes: 41.3% (reference range: 10%-15%); automated eosinophils: 0.09 K/μL (reference range: 0.2-0.8 K/μL); and platelet count: 363 x 10^9/L (reference range: 150-400/L). Also, the albumin test result was 25.8 g/L (reference range: 40.2-47.6 g/L), and the erythrocyte sedimentation rate (ESR) test result was 22 μmol/L (reference range: 3-10 μmol/L). C-reactive protein (CRP) was qualitatively positive. His creatinine test result was 29.1 μmol/L (reference range: 62-115 μmol/L), magnesium 1.02 mmol/L (reference range: 0.7-1.0 mmol/L), and phosphate 2.19 mmol/L (reference range: 0.81-1.58 mmol/L). His blood culture was positive for coagulase-negative staphylococci (CoNS) *(Staphylococcus epidermidis*), which is a gram-positive bacteria.

**Figure 1:**
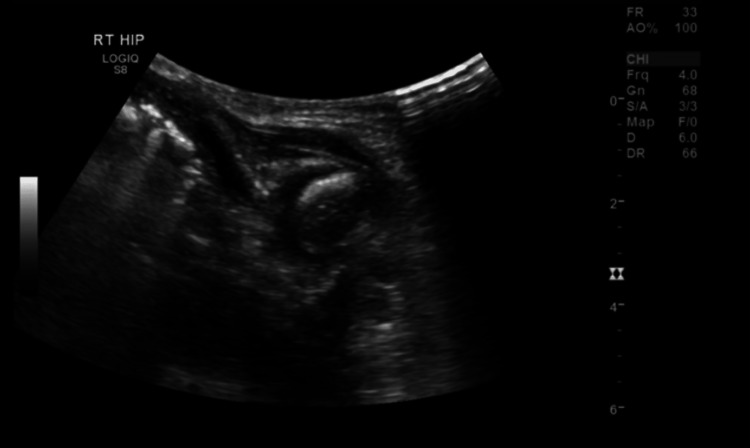
The ultrasound of the right hip showed moderate effusion.

Regarding his radiological studies, including a bilateral hip ultrasound of the patient, it showed unilateral moderate effusion with tiny echoes in the right hip joint (Figure 1).

**Figure 2:**
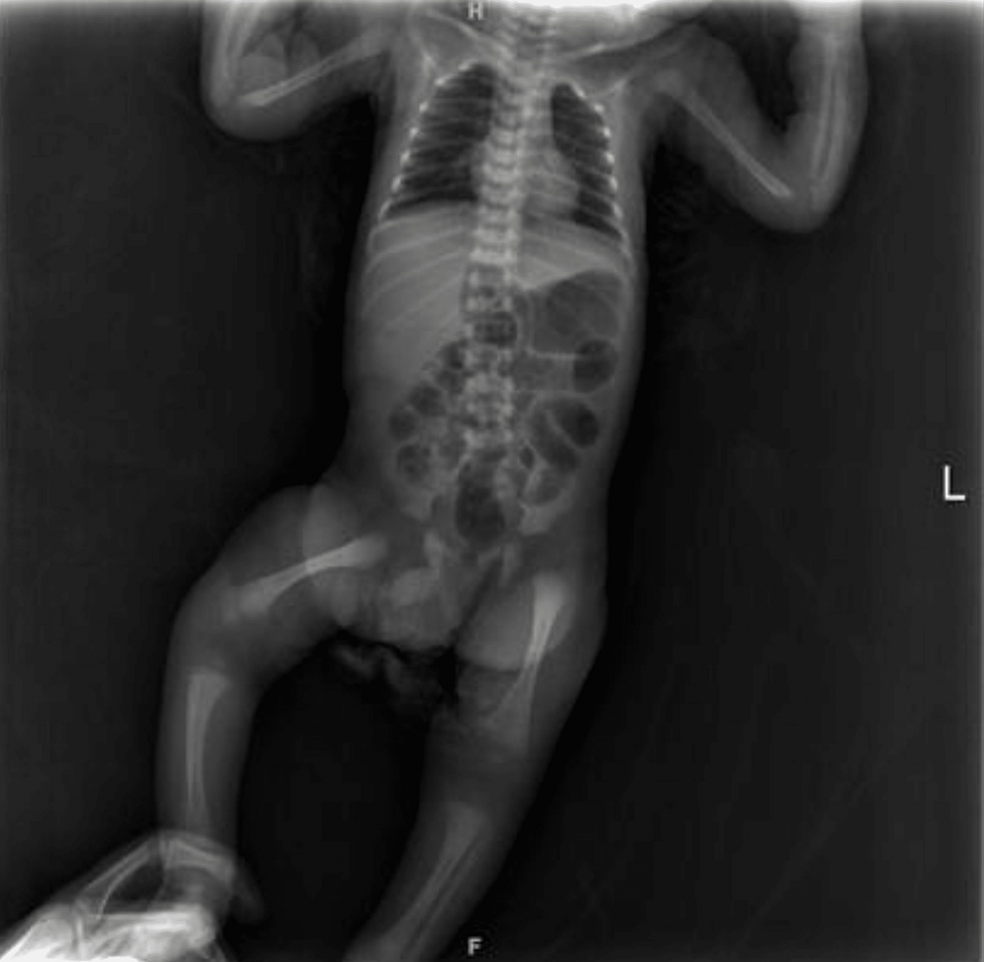
The hip x-ray (anteroposterior (AP) view) showed no findings.

Also, an anteroposterior view of the X-ray was done to rule out osteomyelitis (OM), but there were no findings (Figure 2).

The orthopedic team in the case decided to transfer him to the Maternity and Children Hospital, Makkah, Saudi Arabia, for urgent limb-saving surgical irrigation and debridement (I&D), and a surgical I&D was done on the same day through a Smith-Petersen approach. Plenty of irrigation with normal saline was done, and thick pus was found. The culture sample was taken at that time and sent to the laboratory. After the closure of the capsule, a closed suction drain size 10 was inserted; then consultation with the infectious disease team was done, and their antibiotic plan was: vancomycin (5 mg) and cefotaxime (5 mg), respectively, as empirical treatment from day eight to 18, then culture-guided meropenem (120 mg injection) from day 18 to day 25, and lastly, cefepime (15 mg) and amikacin from day 25 to discharge. Also, there was neither blood collection nor pus coming into the drain, and it was removed after two days.

The patient was admitted postoperatively to the NICU for three days, followed by 27 days of admission in the pediatric ward to complete his antibiotic course. Afterward, the patient was discharged.

**Figure 3:**
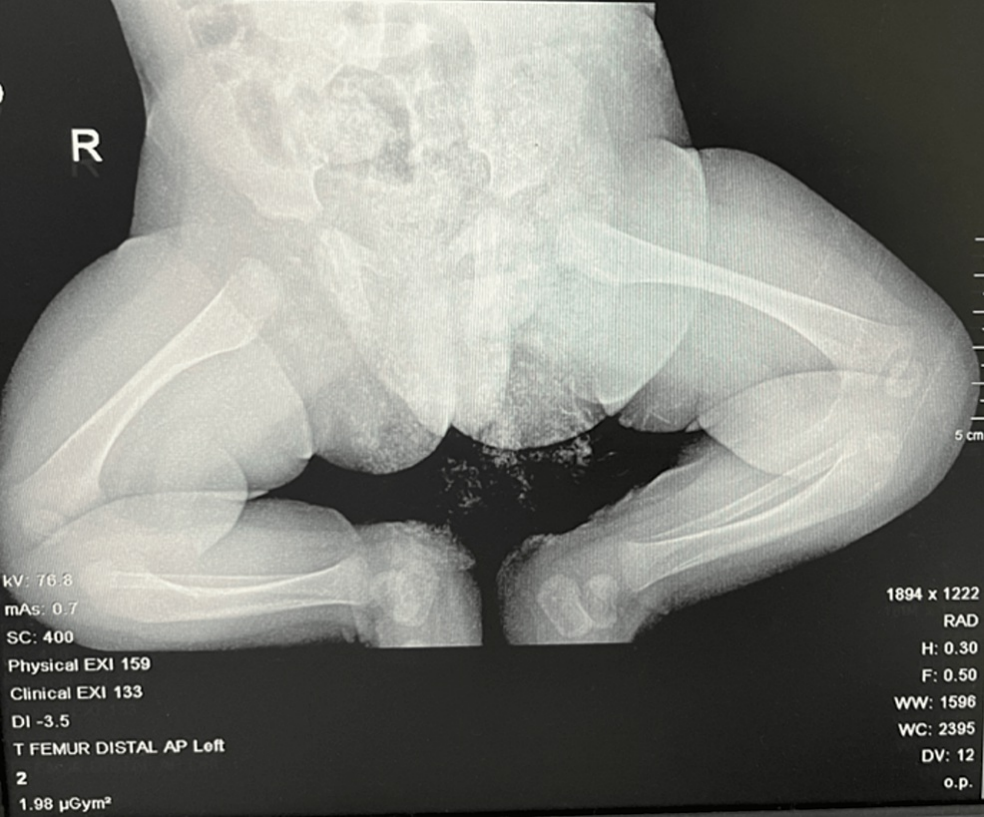
The postoperative hip X-ray (AP view and frog leg lateral view) in an outpatient visit was normal.

At the nine-week follow-up in the clinic, the patient was seen with no fever or active complaint. Upon physical examination, he looked well, conscious, and vitally stable. The scar from surgery was noted, and there was no redness or swelling noted. Also, there was no heat on palpation, and he was actively moving both his lower limbs equally with no restriction, with a normal passive range of motion (ROM) for both hip joints, and the distal neuromuscular examination was intact bilaterally. The inflammatory markers were ordered, and ESR was normal at 10 μmol/L (reference range 3-10 μmol/L). Also, hip X-rays with AP view and frog leg lateral view (Figure 3) were ordered, and they were normal.

## Discussion

Globally, neonatal SA affects around 0.3 out of every 1,000 live births [6], and this number reflects the scarcity of such an illness. However, this incidence may be underreported, especially in neonates, due to a lack of symptoms in the early stages and mild ones, in addition to laboratory results that can be within normal ranges [3].

Similar to our study, a reported case of a six-day-old infant boy presented to the ED after his parents noticed a decrease in the right lower limb movement [3]. Also, a population of 25 neonates with a diagnosis of SA or OM had a mean gestational age of 34.5 weeks (range: 27-40 weeks) and a mean birth weight of 2,269 gms (range: 990-4,750 gms). They reported that the most common presentations were limited mobility (64%) and local swelling (60%) in these neonates [6].

On the other hand, a descriptive study of 29 neonates found that fever was the most common presenting symptom (100%), followed by limb limitation of movement [9]. In our case, fever was not presented, and this can be explained by hypothermia that may develop in early newborns, so the fever is not always present [10]. Therefore, it is crucial to carefully evaluate those patients at this age, in particular, as those patients usually come with vague symptoms and signs such as fever, irritability, and anorexia [6], in which irritability was also presented in our case. Also, because they might merely exhibit irritation, anorexia, and a slight or nonexistent mobility restriction at this age [8], a clinical examination might not be sufficient, and this, as a result, needs certain investigation.

The presenting case was not associated with symptoms and signs of OM, meningitis, or urinary tract infections, as they are considered short-term complications of neonatal SA. On the other hand, some cases were reported previously, and 27% of them had developed avascular necrosis of the femoral head in addition to recurrent dislocation, including the potential for afflicted people to suffer from a permanent handicap [11]. As a result, an early approach to diagnosing SA and appropriate management can prevent these sequelae.

Furthermore, in this case, the patient was a product of CS, which is considered a risk factor for neonatal SA. On the other hand, another study had risk factors other than CS, such as being in respiratory distress syndrome, umbilical artery catheterization, and prematurity [12].

Upon examination in the ED, we found that the patient was vitally stable. The right limb rested in a position of slight flexion, abduction, and external rotation. Also, redness was noticed, and heat was felt upon palpation as well as irritability. Similar to previous cases reported [8, 9],

Additionally, investigations are required as they help identify the infectious agents in all suspected instances of SA of the hip in neonates, in particular a study of the features of joint fluid and culture, and to begin a successful therapy [9]. In our case, the patient's CBC showed elevated leukocyte and neutrophil predominance, similar to the previous studies [8,9,11], and this is commonly suggestive of ongoing bacterial infection [9]. Also, the ESR was elevated, which is an inflammatory marker. Moreover, he has an elevated plasma creatinine level, and this can be explained by a physiological maternal placental transfer that persists in the first two weeks of neonatal life [13].

A blood sample of the patient was sent for culture, and the result showed that it was positive for isolated coagulase-negative staph (*Staphylococcus epidermidis*), which is not the most common causative organism in comparison to previously reported cases in which *Staphylococcus aureus* was the causative organism [6]. We treated the patient by using cefotaxime and vancomycin empirically according to guidelines for any SA to cover the most common organism, and here the nasal swab showed methicillin-resistant *Staphylococcus aureus *(MRSA) [14]. Therefore, we started empirically, and then results appeared from culture-guided Serretia. Gram-negative extended-spectrum β-lactamases (ESBLs), and meropenem worked with it. After meropenem, we changed it into amikacin and cefepime, and they also worked against it.

Also, when a published study reviewed 52 newborn instances of septic arthritis, they cultured *Staphylococcus aureus*, *Klebsiella pneumoniae*, and *Klebsiella oxytoca* from pus or local tissue in 10 patients, three patients, and one child, respectively [11]. We think that these data are related to the limitations and difficulties in joint fluid aspiration and, in general, in microbiological culture in this population.

The diagnosis is made by imaging studies. In our case, a bilateral hip ultrasound (US) was done and showed right hip effusion. Ultrasound is a noninvasive and accurate tool that is frequently used in pediatric imaging, especially in the newborn period [15]. While comparing ultrasound to magnetic resonance imaging (MRI), the latter does not require general anesthesia or sedation and is noninvasive, quick, and radiation-free. Septic arthritis does not require advanced imaging such as MRI prior to arthrotomy and debridement after a hip effusion is verified on ultrasound (US), according to research with a sample of 33 pediatric patients, 26 of whom had a diagnosis of SA alone and six of whom had SA and OM [15]. Furthermore, the management of our case was through arthrotomy for I&D in addition to antibiotics covering the causative organism, similar to previous cases and in contrast to the use of only antibiotics in some cases of late diagnosis [9].

## Conclusions

We have reported a case of septic arthritis that presented early with no complications and was successfully and effectively managed using surgical I&D through the Smith-Petersen approach, in addition to antibiotics. It is necessary to evaluate neonates presenting with vague symptoms and signs from head to toe, as it is significantly important to prevent morbidity and mortality resulting from SA.

## References

[1] Montgomery NI, Epps HR (2017). Pediatric septic arthritis. Orthop Clin North Am.

[2] Erkilinc M, Gilmore A, Weber M, Mistovich RJ (2021). Current concepts in pediatric septic arthritis. J Am Acad Orthop Surg.

[3] Embree JE, Alfattoh NI (2016). Infections in the newborn. Avery’s Neonatology: Pathophysiology and Management of the Newborn (7th Ed).

[4] Rai A, Chakladar D, Bhowmik S (2020). Neonatal septic arthritis: Indian perspective. Eur J Rheumatol.

[5] Kaplan SL (2016). Septic arthritis. Nelson Textbook of Pediatrics. 20th ed.

[6] Narang A, Mukhopadhyay K, Kumar P, Bhakoo ON (1998). Bone and joint infection in neonates. Indian J Pediatr.

[7] Okubo Y, Nochioka K, Marcia T (2017). Nationwide survey of pediatric septic arthritis in the United States. J Orthop.

[8] Gatto A, Lazzareschi I, Onesimo R (2019). Short therapy in a septic arthritis of the neonatal hip. Pediatr Rep.

[9] Sreenivas T, Nataraj AR, Kumar A, Menon J (2016). Neonatal septic arthritis in a tertiary care hospital: a descriptive study. Eur J Orthop Surg Traumatol.

[10] Yitayew YA, Aitaye EB, Lechissa HW, Gebeyehu LO (2020). Neonatal hypothermia and associated factors among newborns admitted in the neonatal intensive care unit of Dessie referral hospital, Amhara region, northeast Ethiopia. Int J Pediatr.

[11] Li Y, Zhou Q, Liu Y (2016). Delayed treatment of septic arthritis in the neonate: a review of 52 cases. Medicine (Baltimore).

[12] Frederiksen B, Christiansen P, Knudsen FU (1993). Acute osteomyelitis and septic arthritis in the neonate, risk factors and outcome. Eur J Pediatr.

[13] Guignard JP, Drukker A (1999). Why do newborn infants have a high plasma creatinine?. Pediatrics.

[14] Chaudhuri A, Martinez-Martin P, Kennedy PG, Andrew Seaton R, Portegies P, Bojar M, Steiner I (2008). EFNS guideline on the management of community-acquired bacterial meningitis: report of an EFNS Task Force on acute bacterial meningitis in older children and adults. Eur J Neurol.

[15] Laine JC, Denning JR, Riccio AI, Jo C, Joglar JM, Wimberly RL (2015). The use of ultrasound in the management of septic arthritis of the hip. J Pediatr Orthop B.

